# Serum luteinizing hormone trajectories during ovarian stimulation and their outcomes of IVF/ICSI: a retrospective cohort study

**DOI:** 10.3389/fendo.2025.1688867

**Published:** 2025-12-09

**Authors:** Conghui Liu, Jiawei Wang, Jing Tian, Shengyuan Chen, Wei Bao, Lei Luo, Limin Wu, Ye Meng

**Affiliations:** 1 Center for Reproduction and Genetics, Department of Gynecology and Obstetrics, The First Affiliated Hospital of University of Science and Technology of China, Division of Life Sciences and Medicine, University of Science and Technology of China, Hefei, Anhui, China; 2 Institute of Public Health Sciences, Division of Life Sciences Medicine, University of Science and Technology of China, Hefei, Anhui, China; 3 Menzies Institute for Medical Research, University of Tasmania, Hobart, TAS, Australia

**Keywords:** luteinizing hormone (LH), trajectories, ovarian stimulation (OS), IVF/ICSI, GnRH antagonists

## Abstract

**Background:**

An appropriate luteinizing hormone (LH) level during ovulation stimulation (OS) is important for positive *in vitro* fertilization/intracytoplasmic sperm injection (IVF/ICSI) outcomes. Although the gonadotrophin-releasing hormone antagonist (GnRH-ant) protocol can inhibit a premature rise of LH, it remains ineffective in some patients. This study aimed to identify the characteristics of patients who shared similar underlying LH trajectories and to determine how different serum LH trajectories influence IVF/ICSI outcomes.

**Methods:**

This study was a retrospective cohort study that included 2,716 patients who underwent GnRH-ant protocol for OS from 1 January 2017 to 31 June 2024. Multiple serum LH measurements during OS were collected. Group-based trajectory modeling was used to identify subgroups of participants who shared similar LH trajectories. Patients’ characteristics and IVF/ICSI outcomes across the identified LH trajectories were compared. Furthermore, linear and log-binomial regression models were used to assess the association of LH trajectories with IVF/ICSI and pregnancy outcomes.

**Results:**

Three discrete LH trajectories were identified: persistently low (90.7%, *n* = 2,464), from middle to high (7.6%, *n* = 206), and up and down (1.7%, *n* = 46). Compared to patients with persistently low trajectory, a higher number of antral follicle counts (AFC) and level of basal LH were observed for patients with middle to high and up and down trajectories. Although a higher number of oocytes and embryos were found for patients with middle to high and up and down trajectories than those with persistently low trajectory, metaphase II oocytes and embryo formation rates were lower. Despite these differences, pregnancy outcomes after fresh embryo transfer were similar across the three trajectories.

**Conclusions:**

Patients with an elevated number of AFC and basal LH were more likely to exhibit unstable LH trajectories. Higher LH trajectories during ovarian stimulation were associated with a better quantity rather than the quality of oocytes or embryos.

## Introduction

Luteinizing hormone (LH) is essential for follicular development and oocyte maturation (1–4). Variations in LH level during the follicular phase impact the morphology and function of oocytes, thereby affecting meiotic status and fertilization ability (1, 3). Moreover, a surge of LH triggers ovulation and prompts the production of progesterone by the corpus luteum, which is critical for endometrial receptivity (5). Previous studies have indicated that both excessively high and insufficient LH levels were associated with adverse outcomes of *in vitro* fertilization/intracytoplasmic sperm injection (IVF/ICSI) (6–8). Elevated LH concentrations may impair endometrial receptivity, and a low LH level that does not meet the LH threshold might block paracrine signaling between granulosa and theca and restrain the synthesis of androgen and oocyte maturation (9, 10). Therefore, maintaining an appropriate serum LH level during ovulation stimulation is crucial for achieving positive IVF/ICSI and pregnancy outcomes.

The gonadotrophin-releasing hormone antagonist (GnRH-ant) protocol, which has been widely used in recent decades, is one of the most important protocols for ovarian stimulation of IVF/ICSI. Compared with traditional ovarian stimulation protocols [e.g., gonadotrophin-releasing hormone agonist (GnRH-a) short protocol, GnRH-a long protocol, etc.], the GnRH-ant protocol has several advantages: 1) a shorter duration of ovarian stimulation, 2) a reduced dosage of gonadotropic (Gn) hormones, and 3) a lower risk of ovarian hyperstimulation syndrome (OHSS) (11). Although the GnRH-ant protocol effectively prevents a premature rise of LH during COS by competitively binding to GnRH receptors following immediate pituitary suppression, some patients still experience a premature rise of LH despite antagonist administration. Furthermore, for patients who were sensitive to GnRH antagonist, serum levels of LH may be over-suppressed, necessitating exogenous LH supplementation (12). Although several studies have suggested that a premature rise of LH level during ovarian stimulation would result in poor embryo quality and low pregnancy rate (13, 14), the association between serum LH during ovarian stimulation and IVF/ICSI outcomes is still controversial. Up to now, no study has investigated the trajectory of serum LH continuously, and the characteristics of patients who were more likely to have an unstable LH trajectory are still unknown, as well as the relationship between these dynamic LH trajectories and IVF/ICSI and pregnancy outcomes.

To fill these gaps, this study aimed to identify the characteristics of patients who shared similar underlying LH trajectories during ovarian stimulation and investigate the influence of different serum LH trajectories on IVF/ICSI and pregnancy outcomes.

## Materials and methods

### Study population

This was a retrospective cohort study designed to examine the effect of serum LH trajectories during ovarian stimulation on oocyte retrieval, fertilization, and embryo development of IVF/ICSI. All GnRH-ant cycles performed at the Reproduction and Genetics Center of the First Affiliated Hospital of the University of Science and Technology of China (USTC) from 1 January 2017 to 31 June 2024 were assessed. A total of 2,716 patients meeting the inclusion and exclusion criteria for their ovarian stimulation cycle were included. The inclusion criteria were as follows: 1) patients younger than 35 years old, 2) patients with a body mass index (BMI) less than 28 kg/m^2^, 3) patients with an antral follicle count (AFC) of seven or more, and 4) patients who underwent GnRH-ant protocol for their ovarian stimulation. The exclusion criteria were 1) patients with bilateral or unilateral hydrosalpinx detected by ultrasound or hysterosalpingography, 2) patients with uterine diseases (e.g., intrauterine adhesions, submucosal fibroids, endometrial polyps, etc.), and 3) patients who underwent an ovarian stimulation protocol other than a GnRH-ant protocol. Basic information included age, BMI, the levels of basal serum LH, follicle-stimulating hormone (FSH), estradiol (E_2_), and duration and total dose of gonadotrophin (Gn) administered. IVF/ICSI outcomes included the number of oocytes retrieved, metaphase II (MII) oocytes, 2 pronuclear (2PN) fertilized oocytes, good-quality day-3 embryos, blastocyst, and good-quality blastocyst, and pregnancy outcomes were collected from medical records. This study was approved by the Reproductive Medicine Ethics Committee of the First Affiliated Hospital of USTC (No. 2024-RE-427).

### Controlled ovarian stimulation

Ovarian stimulation began on day 2 or 3 of the menstrual cycle with recombinant follicle-stimulating hormone (FSH, Bravelle^®^; Ferring Pharmaceuticals Inc.) 150–300 IU daily. The starting dose of Gn was based on the patient’s age, hormone profile, AFC, and BMI. Doses of Gn were adjusted according to serum hormone profile including LH, FSH, E_2_, and progesterone (measured every 2–5 days when follicles <16 mm and daily when follicles ≥16mm) and ovarian response (evaluated by transvaginal ultrasound). All the serum hormones were measured by chemiluminescent immunoassay using Siemens Atellica IM Analyzer according to the manufacturer’s instructions. GnRH-antagonist (0.25 mg/day) was initiated when the lead follicle reached 12 to 14 mm in diameter or a rise in LH and was continued until the day of hCG administration. When there were three follicles >17 mm or two follicles >18 mm, 250 μg of recombinant human chorionic gonadotrophin (rHCG; Merck, Germany) or 0.1/0.2 IU of triptorelin (Ferring International Center, Saint-Prex, Switzerland) with 4,000–10,000 IU of hCG (Livzon, Zhuhai, China) was administered. Oocyte retrieval was performed by transvaginal ultrasonography 36–38 h later.

### *In vitro* fertilization and embryo quality evaluation

All retrieved oocytes were fertilized with either conventional IVF/ICSI 2–4 h after oocyte retrieval. Only patients experiencing severe male factor infertility underwent ICSI. Fertilization was assessed 16–18 h after insemination for the appearance of two distinct pronuclei and two polar bodies. The zygotes were cultured in a cleavage medium (Vitrolife, Sweden). Embryonic development was assessed daily. Characteristics of good embryos on day 3 were those evaluated as grade I and grade II embryos (grade I: a. display eight cells; b. the size of blastomere is uniform; c. homogeneous cytoplasm; d. fragments less than 5.0%; grade II: a. display six to eight cells; b. the size of the blastomere is uniform or uneven; c. fragments less than 20.0%). Most patients (89.0%) had their embryos cultured to the blastocyst stage in blastocyst medium (Vitrolife). Blastocyst quality scoring was performed on day 5 according to Gardner’s criteria (15). Characteristics of good blastocysts were those evaluated as grade I and grade II embryos (grade 1: AA; grade II: AB, BA, and BB) (16). All the scoring was conducted by two independent, senior embryologists.

During fresh embryo transfer cycles, embryos were transferred on day 3 or day 5 after fertilization. All embryos would be frozen if a patient had issues related to a thin endometrial lining (<7 mm), intrauterine fluid, hydrosalpinx, elevated progesterone level (>1.0 ng/mL) on the day of hCG administration (17), or had a high risk of OHSS.

### Statistical analyses

Group-based trajectory modeling (GBTM) was used to identify subgroups of participants who shared similar underlying LH trajectories. All trajectories were identified by the Traj plugin in Stata software 17.0 (StataCorp, College Station, Texas). A detailed description of GBTM can be found elsewhere (18). The optimal number (ranging from 2 to 4) and the best model of LH trajectories (linear, quadratic, or cubic) were determined according to Bayesian information criteria (BIC), Akaike information criterion (AIC), average posterior probability (AvePP), proportion of participants with a posterior probability ≥70.0%, and biological plausibility. Lower values of BIC (primary statistical criterion) and AIC indicate a better balance between model fit and parsimony. Posterior probability is the likelihood that an individual belongs to a specific trajectory, with higher AvePP indicating greater certainty in classification. In our study, there were 98.5%, 82.5%, and 89.1% patients in the three trajectories, respectively, with posterior probability ≥70.0%. A total of 2,716 patients who had a baseline LH measurement on the first day and more than two additional LH measurements during the subsequent ovarian stimulation were included in the analyses to identify LH trajectories (19). Finally, three distinct LH trajectories were determined as the best-fitting model, as shown in **Supplementary Table 1**.

Means with standard deviations were reported to summarize continuous variables, and proportions with numbers were reported to summarize categorical variables. Comparisons of characteristics across the identified LH trajectories were performed using one-way ANOVA for continuous variables when the data were normally distributed with homogeneity of variance; otherwise, the Kruskal–Wallis test was used. The *χ*^2^ test was used for categorical variables. The LSD test or Bonferroni correction was used for pairwise comparisons.

Linear regression models were used to assess the association of LH trajectories with the numbers of oocytes received, MII oocytes, 2PN fertilized oocytes, day-3 embryos, blastocyst, and good-quality blastocyst. Log-binomial regression models were used to determine relative risks (RRs) and 95% confidence intervals (CI) for the associations between LH trajectories and pregnancy outcomes. The confounders adjusted in our regression models were causally associated with the outcome according to the literature, imbalanced between the exposure groups, not mediators between the exposure and outcome, and resulted in more than 10% variation in the coefficient of the study factor when added into regression models.

Sensitivity analyses restricting analyses to participants with posterior probability ≥70% were added in **Supplementary Tables 2–5** to check the robustness of our results.

A two-tailed *P-*value of less than 0.05 was considered statistically significant. All analyses were performed with STATA version 17.0 (StataCorp, College Station, Texas).

## Results

### Characteristics of LH trajectories and participants

**Figure 1:**
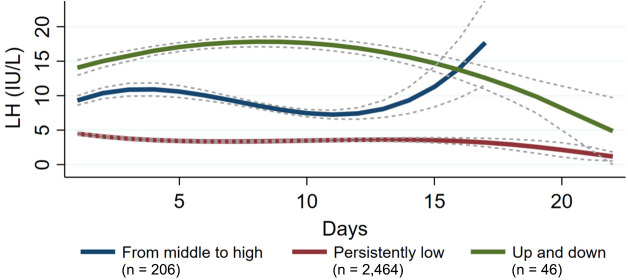
Group-based trajectories for luteinizing hormone (LH) during ovarian stimulation, *n* = 2,716.

A total of 2,716 patients who met the inclusion criteria were included in the analyses, with an average of 9 days of ovarian stimulation. Based on GBTM, three discrete LH trajectories were identified: persistently low, middle to high, and up and down. As shown in **Figure 1**, 90.7% of patients had a serum LH level that remained low relative to others throughout the ovarian stimulation (persistently low, *n* = 2,464); 7.6% of patients had a moderate serum LH level compared to others, presenting a rise at the end of the ovarian stimulation (from middle to high, *n* = 206); and 1.7% of patients showed an unstable LH level throughout the ovarian stimulation (up and down, *n* = 46).

The characteristics of patients in total and by identified LH trajectories are shown in **Table 1**. The mean age of the total patients was 29.6 years, with an average BMI of 22.3 kg/m^2^. Most oocytes were fertilized by IVF rather than ICSI (90.8% vs. 9.2%). Compared to patients with persistently low trajectory, a higher number of AFC (18.3 *vs*. 22.0 *vs*. 21.7, *P* < 0.001) with a higher level of basal LH (4.6 *vs*. 8.0 *vs*. 9.4 mIU/mL, *P* < 0.001) were observed for patients with middle to high and up and down trajectories. Moreover, patients with persistently low trajectory were older (29.7 *vs*. 29.0 *vs*. 29.2 years old, *P* = 0.002) and more likely to have a history of pregnancy (40.5% *vs*. 34.0% *vs*. 21.7%, *P* = 0.01) compared to the other two trajectories. Other characteristics, including basal FSH, E_2_, duration of infertility, and fertilization way, were similar across the three trajectories.

**Table 1:** Characteristics of patients on different LH trajectories during ovarian stimulation (*n* = 2,716).

Characteristics	Total patients	Persistently low	From middle to high	Up and down	*P*-value
*N*	2,716	2,464	206	46	
Age (years), mean (SD)	29.6 (3.0)	29.7 (3.0)^*^	29.0 (3.0)^*^	29.2 (3.2)	**0.002**
BMI (kg/m^2^), mean (SD)	22.3 (2.7)	22.3 (2.7)	22.6 (2.7)	22.8 (2.3)	0.17
Duration of infertility (years), mean (SD)	2.7 (1.9)	2.7 (1.9)	2.5 (1.5)	2.8 (1.6)	0.14
Infertility type, % (*n*)					**0.01**
Primary infertility	60.4 (1,639)	59.5 (1,467)^*^	66.0 (136)	78.3 (36)^*^	
Secondary infertility	39.7 (1,077)	40.5 (997)	34.0 (70)	21.7 (10)	
Basal serum FSH (mIU/mL), mean (SD)	6.4 (4.7)	6.4 (4.5)	6.8 (7.0)	6.9 (3.6)	0.32
Basal serum LH (mIU/mL), mean (SD)	5.0 (4.6)	4.6 (4.4)^*,#^	8.0 (5.4)^*^	9.4 (6.3)^#^	**<0.001**
Basal serum E_2_ (pg/mL), mean (SD)	48.2 (45.1)	47.9 (46.4)	50.6 (29.0)	51.7 (32.8)	0.21
AFC, mean (SD)	18.6 (6.5)	18.3 (6.4)^*,#^	22.0 (5.9)^*^	21.7 (6.9)^#^	**<0.001**
Fertilization way, % (*n*)					0.30
IVF	90.8 (2,465)	92.7 (191)	90.5 (2,230)	95.7 (44)	
ICSI	9.2 (251)	7.3 (15)	9.5 (234)	4.4 (2)	

*n*, number; SD, standard deviation; BMI, body mass index; FSH, follicle-stimulating hormone; LH, luteinizing Hormone; E_2_, estrogen; AFC, antral follicle count; IVF, *in vitro* fertilization; ICSI, intracytoplasmic sperm injection.

^*,#^The same superscript in a row indicates statistically significant differences.Bold value indicates statistically significant differences.

### Oocyte and embryo outcomes

As shown in **Tables 2**, **3**, patients with persistently low trajectory used a greater amount of total Gn (2,004.2 IU) than those with middle to high (1,716.9 IU) and up and down (1,587.0 IU) trajectories. Compared with patients with persistently low trajectory, a significantly higher number of received oocytes (*β* = 3.80 [95% CI: 2.45 to 5.15] for patients with middle to high trajectory and *β* = 4.19 [95% CI: 0.97 to 7.41] for patients with up and down trajectory), MII oocytes (*β* = 3.08 [95% CI: 1.89 to 4.27] for patients with middle to high trajectory and *β* = 3.87 [95% CI: 0.90 to 6.84] for patients with up and down trajectory), 2PN fertilized oocytes (*β* = 2.07 [95% CI: 1.13 to 3.01] for patients with middle to high trajectory and *β* = 2.73 [95% CI: 0.42 to 5.03] for patients with up and down trajectory), and day-3 embryos (*β* = 2.25 [95% CI: 1.28 to 3.22] for patients with middle to high trajectory and *β* = 2.96 [95% CI: 0.57 to 5.35] for patients with up and down trajectory) were observed in patients with middle to high and up and down trajectories. Meanwhile, the rate of MII oocytes for patients with middle to high trajectory was the least among the three trajectories (86.5% *vs*. 88.0% *vs*. 89.1%). Although the average number of oocytes received was 17.5 for patients with up and down trajectory, the average number of good-quality day 3 embryos was only 5.6 with a formation rate of 36.0%, which is the lowest among the three trajectories (40.2% for the persistently low trajectory; 41.5% for the middle to high trajectory).

**Table 2:** The cycle and embryo characteristics of patients on different LH trajectories during ovarian stimulation.

Characteristics	Total patients	Persistently low	From middle to high	Up and down	*P*-value
*n*	2,716	2,464	206	46	
Total dose of Gn (IU), mean (SD)	1,975.3 (985.3)	2,004.2 (988.6)^*,#^	1,716.9 (911.6) ^*^	1,587.0 (885.7)^#^	**<0.001**
Duration of ovarian stimulation (days), mean (SD)	9.9 (2.0)	9.9 (2.0)	9.8 (1.8)	9.9 (3.3)	0.49
MII oocytes rate, % (*n*)	87.9 (32,082)	88.0 (28,297)^*^	86.5 (3,066)^*,#^	89.1 (719)^#^	**0.02**
2PN fertilized rate, % (*n*)	73.7 (23,640)	73.9 (20,898)	72.3 (2,218)	72.9 (524)	0.17
Embryo formation rate, % (*n*)	74.9 (24,041)	75.0 (21,216)	74.5 (2,284)	75.2 (541)	0.83
Good-quality day-3 embryo rate, % (*n*)	40.3 (12,918)	40.2 (11,388)^*^	41.5 (1,271)^#^	36.0 (259)^*,#^	**0.03**
Blastocyst culture, *n*	2,373	2,146	190	37	
Blastocyst formation rate, % (*n*)	51.4 (9,199)	51.4 (8,084)	52.2 (921)	49.1 (194)	0.53
Good-quality blastocyst formation rate, % (*n*)	37.1 (6,633)	37.3 (5,867)^*^	36.4 (642)	31.4 (124)^*^	**0.047**
Elevated serum levels of P on trigger day, % (*n*)	10.6 (236)	10.2 (204)^*^	12.9 (23)	22.5 (9)^*^	**0.01**

Gn, gonadotropin-releasing hormone; *n*, number; SD, standard deviation; MII, metaphase II; 2PN, 2 pronuclear; ET, embryo transfer; P, progesterone.

^*,#^The same superscript in a row indicates statistically significant differences.Bold value indicates statistically significant differences.

**Table 3:** Association of LH trajectories with IVF/ICSI and embryo outcomes.

LH trajectory group	% (*n*)	Unadjusted	Adjusted^*^
		*β* (95% CI)	*β* (95% CI)
No. of oocytes received			
Persistently low	13.0 (7.8)	Reference	Reference
From middle to high	17.2 (9.9)	**4.17 (2.79 to 5.54)**	**3.80 (2.45 to 5.15)**
Up and down	17.5 (10.8)	**4.50 (1.39 to 7.61)**	**4.19 (0.97 to 7.41)**
No. of MII oocytes			
Persistently low	11.5 (7.1)	Reference	Reference
From middle to high	14.9 (8.6)	**3.40 (2.19 to 4.61)**	**3.08 (1.89 to 4.27)**
Up and down	15.6 (10.0)	**4.15 (1.26 to 7.03)**	**3.87 (0.90 to 6.84)**
No. of 2PN fertilized oocytes			
Persistently low	8.5 (5.6)	Reference	Reference
From middle to high	10.8 (6.8)	**2.29 (1.34 to 3.23)**	**2.07 (1.13 to 3.01)**
Up and down	11.4 (7.8)	**2.91 (0.66 to 5.16)**	**2.73 (0.42 to 5.03)**
No. of day-3 embryos			
Persistently low	8.6 (5.7)	Reference	Reference
From middle to high	11.1 (7.0)	**2.48 (1.50 to 3.46)**	**2.25 (1.28 to 3.22)**
Up and down	11.8 (8.1)	**3.15 (0.82 to 5.49)**	**2.96 (0.57 to 5.35)**
No. of good-quality day-3 embryos			
Persistently low	4.6 (4.2)	Reference	Reference
From middle to high	6.2 (5.1)	**1.55 (0.83 to 2.26)**	**1.43 (0.71 to 2.14)**
Up and down	5.6 (5.8)	1.01 (−0.65 to 2.67)	0.90 (−0.77 to 2.57)
No. of blastocyst culture			
Persistently low	7.3 (5.3)	Reference	Reference
From middle to high	9.3 (6.3)	**1.96 (1.03 to 2.88)**	**1.79 (0.88 to 2.70)**
Up and down	10.7 (7.4)	**3.34 (0.98 to 5.70)**	**3.20 (0.80 to 5.60)**
No. of blastocyst			
Persistently low	3.8 (3.6)	Reference	Reference
From middle to high	4.8 (4.0)	**1.08 (0.49 to 1.67)**	**1.01 (0.42 to 1.60)**
Up and down	5.2 (4.6)	1.48 (0 to 2.96)	1.42 (−0.08 to 2.92)
No. of good-quality blastocyst			
Persistently low	2.7 (3.0)	Reference	Reference
From middle to high	3.4 (3.2)	**0.65 (0.18 to 1.11)**	**0.60 (0.13 to 1.07)**
Up and down	3.4 (3.3)	0.62 (−0.42 to 1.66)	0.58 (−0.47 to 1.64)

*n* or No., number; CI, confidence interval; MII, metaphase II; 2PN, 2 pronuclear; AFC, antral follicle count; BMI, body mass index.

^*^Adjusted for age and BMI.Bold value indicates statistically significant differences.

In addition, 2,146 out of 2,464 patients with a mean number of 7.3 cleavage embryos with persistently low trajectory, 190 out of 206 patients with a mean number of 9.3 cleavage embryos with middle to high trajectory, and 37 out of 46 patients with a mean number of 10.7 cleavage embryos with up and down trajectory underwent blastocyst culture. Compared with patients with persistently low trajectory, the number of blastocysts was higher for patients with middle to high trajectory (*β* = 1.01, 95% CI: 0.42 to 1.60). No significant difference was found on blastocyst formation rate among the three groups (51.4% for persistently low, 52.2% for middle to high, and 49.1% for up and down; *P* = 0.53).

**Table 2** also shows that 10.2% of patients with persistently low trajectory had an elevated serum level of progesterone on trigger day, and the rate for patients with middle to high trajectory was 12.9%, while the rate doubled for patients with up and down trajectory (22.5%).

### Pregnancy outcomes

As shown in **Table 4**, the rates of biochemical pregnancy, clinical pregnancy, and uterine pregnancy were similar across the three trajectories for all embryo transfer cycles, blastocyst transfer cycle, and cleavage embryo transfer cycle. For all embryo transfer cycles, the clinical pregnancy rate was 48.2% for patients with persistently low trajectory, 50.0% for patients with middle to high trajectory, and 33.3% for patients with up and down trajectory, and the rates for blastocyst transfer cycle were 50.3%, 48.4%, and 20.0%, respectively. Although clinical pregnancy rates for the cleavage embryo transfer cycle were 38.3%, 66.7%, and 100.0%, respectively, only three patients with middle to high trajectory and one patient with up and down trajectory underwent cleavage embryo transfer. Furthermore, regression analyses also show that there were no significant associations between LH trajectories and pregnancy outcomes before and after adjustment after fresh blastocyst transfer (**Table 5**).

**Table 4:** Pregnancy outcomes among patients who underwent fresh embryo transfer cycle with different LH trajectories during ovarian stimulation.

Pregnancy outcomes	Total patients	Persistently low	From middle to high	Up and down	*P*-value
All embryo transfer, *n*	702	662	34	6	
Biochemical pregnancy rate, % (*n*)	58.7 (412)	58.9 (390)	58.8 (20)	33.3 (2)	0.45
Clinical pregnancy rate, % (*n*)	48.2 (338)	48.2 (319)	50.0 (17)	33.3 (2)	0.75
Uterine pregnancy rate, % (*n*)	97.6 (330)	97.5 (311)	100.0 (17)	100.0 (2)	0.98
Blastocyst transfer, *n*	583	547	31	5	
Biochemical pregnancy rate, % (*n*)	60.4 (352)	60.9 (333)	58.1 (18)	20.0 (1)	0.17
Clinical pregnancy rate, % (*n*)	49.9 (291)	50.3 (275)	48.4 (15)	20.0 (1)	0.40
Uterine pregnancy rate, % (*n*)	97.6 (284)	97.5 (268)	100.0 (15)	100.0 (1)	0.42
Cleavage embryo transfer, *n*	119	115	3	1	
Biochemical pregnancy rate, % (*n*)	50.4 (60)	49.6 (57)	66.7 (2)	100.0 (1)	0.51
Clinical pregnancy rate, % (*n*)	39.5 (47)	38.3 (44)	66.7 (2)	100.0 (1)	0.28
Uterine pregnancy rate, % (*n*)	97.9 (46)	97.7 (43)	100.0 (2)	100.0 (1)	0.97

*n*, number; LH, luteinizing hormone.

**Table 5:** Association of LH trajectories with pregnancy outcomes among patients who underwent fresh blastocyst transfer.

LH trajectory group	Unadjusted	Adjusted^*^
	RR (95% CI)	RR (95% CI)
Biochemical pregnancy		
Persistently low	Reference	Reference
From middle to high	0.95 (0.70–1.30)	0.94 (0.69–1.28)
Up and down	0.33 (0.06–1.90)	0.31 (0.05–1.79)
Clinical pregnancy		
Persistently low	Reference	Reference
From middle to high	0.96 (0.66–1.40)	0.96 (0.66–1.39)
Up and down	0.40 (0.07–2.30)	0.38 (0.07–2.20)

RR, relative risk; CI, confidence interval; AFC, antral follicle count; BMI, body mass index.

^*^Adjusted for age and BMI.

Sensitivity analyses were performed by only including patients with posterior probabilities >70% in the identified trajectories, and the results were similar to the main analyses (**Supplementary Tables 2–5**).

## Discussion

This study is the first to identify the characteristics of patients who shared similar underlying LH trajectories and examine the influence of different serum LH trajectories on IVF/ICSI outcomes. Three distinct LH trajectories were established: persistently low trajectory, middle to high trajectory, and up and down trajectory. Patients with a higher number of AFC and basal LH were more likely to have an unstable LH trajectory. Findings between serum LH trajectories and IVF/ICSI outcomes indicated that although patients with higher LH trajectories (from middle to high and up and down trajectories) had a higher number of MII oocytes and embryos compared with patients with persistently low LH trajectory, the rates of MII oocytes or good-quality embryos were still lower. Therefore, higher serum LH trajectories during ovarian stimulation were associated with a better quantity rather than the quality of oocytes and embryos.

LH plays a critical role in follicular development (3, 20). According to the “two-cell, two-gonadotrophin” theory of ovarian function, LH stimulates the theca-interstitial cell layer to produce androgens, which then act as substrates for estradiol synthesis in granulosa cells via a paracrine mechanism (21, 22). This process promotes the transition of follicles from the early to the antral stage, thereby contributing to the functional ovarian reserve (23). La Marca et al. (24) reported that the administration of LH for patients with hypothalamic amenorrhea and low functional ovarian reserve may increase the number of their AFC. Our study aligns with this theory and found that patients with a higher LH trajectory during ovarian stimulation had a higher number of AFC and a higher level of basal LH. Furthermore, our study adds evidence that excessive basal LH levels may result in an unstable LH trajectory during ovarian stimulation.

In nature, LH surges approximately 28 to 36 h before ovulation. Although the GnRH-ant protocol can inhibit a premature rise of LH during ovarian stimulation by competitively binding to GnRH receptors after immediate pituitary suppression, several patients still experience an early-onset LH surge. This early LH surge may adversely affect both oocyte/embryo quality and pregnancy outcomes (13, 25–27). Previous studies have suggested that elevated basal LH levels, particularly in patients with increased AFC such as those with PCOS, may raise the risk of early-onset LH surge, although other studies have contested this view (28, 29). Recently, the ratio of serum LH level on the trigger day to the basal serum LH level has emerged as a potential predictor for evaluating the impact of serum LH on embryo development potential and pregnancy outcomes (30–32). However, no study has yet dynamically examined serum LH levels from the basal stage to the trigger day or systematically assessed their influence on IVF/ICSI outcomes. In our study, three distinct serum LH trajectories were identified. Among them, patients with up and down LH trajectory had the highest numbers of AFC and levels of basal serum LH, and an early-onset LH surge was indeed observed within this trajectory.

In terms of the use of Gn, although patients with up and down LH trajectory used the least amount of total Gn (1,587.0 IU) and collected the highest numbers of day-3 embryos, their embryo formation rate was only 36.0%, indicating impaired follicle quality in this group. Moreover, these patients also exhibited the highest incidence of elevated serum progesterone on the trigger day, suggesting that unstable serum LH during ovarian stimulation may prompt progesterone production. Further studies are needed to determine the optimal time for antagonist implementation to prevent premature LH rise in patients with different characteristics, particularly those with higher numbers of AFC and levels of basal serum LH.

A previous randomized controlled trial involving 213 patients undergoing GnRH-ant protocol for ovarian stimulation revealed that a lower trend in LH values during ovarian stimulation was associated with a higher pregnancy rate (6). However, LH levels were measured only on the first day of stimulation, the day following GnRH-ant administration, and the day of hCG administration (6). Similarly, a retrospective study by Zhang et al. (33) reported that patients with serum LH levels below 2.6 mIU/mL on the trigger day had higher rates of embryo implantation, clinical pregnancy, and live birth than those with serum LH levels above 2.6 mIU/mL, although this study was conducted exclusively in patients with diminished ovarian reserve. In the same year, Chen et al. (34) observed similar findings in patients with normal ovarian function. Moreover, Xu et al. (35) believed that serum LH levels exceeding 2.0 mIU/mL on the trigger day were associated with better embryo quality and implantation rates. Up to now, previous studies have primarily focused on the impact of serum LH levels at single or a few specific time points, rather than examining the dynamic trajectories of LH throughout the entire ovarian stimulation process. Based on GBTM, our study continuously assessed serum LH trajectories and identified three distinct LH trajectories. We found that patients with middle to high LH trajectory had the highest rate of good-quality day-3 embryos, suggesting that both too-high and too-low LH levels during ovarian stimulation may deteriorate the quality of follicles and embryos.

Our study underscores the importance of enhancing the monitoring of serum LH levels during ovarian stimulation for patients with a higher number of AFC and basal LH, which allows timely medication adjustments and trigger administration. For patients who had higher numbers of embryos but experience unstable LH trajectories, blastocyst culture serves as a valuable strategy to select high-quality, developmentally competent embryos for transfer, thereby improving pregnancy rates in IVF/ICSI cycles. Further studies are needed to identify a reasonable, achievable frequency for serum LH testing during ovarian stimulation, aiming to enhance both embryo quantity and quality. Additionally, future studies should investigate whether a threshold effect exists whereby a greater number of embryos can compensate for reduced overall embryo quality.

There are several limitations to the study. First, the retrospective design had inherent problems related to selection bias (36), whereas most characteristics including the duration of infertility, infertility type, basal FSH, basal E_2_, and fertilization way were similar among patients with the three trajectories. Second, as all participants were recruited from a single center, the generalizability of the findings may be limited; thus, the results should be interpreted with caution. Further studies from multiple hospitals with larger sample size should be carried out to confirm our findings. Third, the number of patients in the “from middle to high” and “up and down” LH trajectory groups was relatively small, which aligns with the physiological pattern of the LH surge typically occurring 28–36 h before ovulation. Nevertheless, the consistency between the sensitivity analyses and the primary findings supports the robustness of the results. Finally, since fresh embryo transfers were canceled for patients with elevated progesterone levels on the trigger day, we could not evaluate the potential influence of LH trajectories on endometrial receptivity and subsequent pregnancy outcomes.

There are also strengths to our study. The multiple tests of serum LH levels during the ovarian stimulation allowed us to identify heterogeneity in LH trajectories. To our knowledge, this is the first study to characterize distinct patient subgroups based on underlying LH trajectory patterns and to assess their associations with IVF/ICSI outcomes. Moreover, critical inclusion and exclusion criteria in our study minimized the heterogeneity of patients included in this study. In addition, a range of covariates available in the study allowed us to consider potential confounding.

## Conclusions

In conclusion, this study identified three discrete LH trajectories during ovarian stimulation: persistently low trajectory, middle to high trajectory, and up and down trajectory. Patients with a higher number of AFC and levels of basal LH were more likely to exhibit an unstable LH trajectory. Higher serum LH trajectories during ovarian stimulation were associated with a better quantity rather than the quality of oocytes and embryos.

## Data Availability

The raw data supporting the conclusions of this article will be made available by the authors, without undue reservation.

## References

[1] Huirne JA Lambalk CB . Gonadotropin-releasing-hormone-receptor antagonists. Lancet. (2001) 358:1793–803. doi: 10.1016/s0140-6736(01)06797-6, PMID: 11734258

[2] Filicori M . The role of luteinizing hormone in folliculogenesis and ovulation induction. Fertil Steril. (1999) 71:405–14. doi: 10.1016/s0015-0282(98)00482-8, PMID: 10065772

[3] Arroyo A Kim B Yeh J . Luteinizing hormone action in human oocyte maturation and quality: signaling pathways, regulation, and clinical impact. Reprod Sci. (2020) 27:1223–52. doi: 10.1007/s43032-019-00137-x, PMID: 32046451 PMC7190682

[4] Toner JP Pirtea P . Luteinizing hormone's critical role in ovarian stimulation. Fertil Steril. (2025) 123:31–40. doi: 10.1016/j.fertnstert.2024.11.005, PMID: 39522745

[5] Gizzo S Andrisani A Noventa M Manfè S Oliva A Gangemi M . Recombinant LH supplementation during IVF cycles with a GnRH-antagonist in estimated poor responders: A cross-matched pilot investigation of the optimal daily dose and timing. Mol Med Rep. (2015) 12:4219–29. doi: 10.3892/mmr.2015.3904, PMID: 26059981 PMC4526099

[6] Depalo R Trerotoli P Chincoli A Vacca MP Lamanna G Cicinelli E . Endogenous luteinizing hormone concentration and IVF outcome during ovarian stimulation in fixed versus flexible GnRH antagonist protocols: An RCT. Int J Reprod Biomed. (2018) 16:175–82. doi: 10.29252/ijrm.16.3.175, PMID: 29766148 PMC5944439

[7] Bosch E Escudero E Crespo J Simón C Remohí J Pellicer A . Serum luteinizing hormone in patients undergoing ovarian stimulation with gonadotropin-releasing hormone antagonists and recombinant follicle-stimulating hormone and its relationship with cycle outcome. Fertil Steril. (2005) 84:1529–32. doi: 10.1016/j.fertnstert.2005.05.040, PMID: 16275263

[8] Balasch J Fábregues F . LH in the follicular phase: neither too high nor too low. Reprod BioMed Online. (2006) 12:406–15. doi: 10.1016/s1472-6483(10)61991-8, PMID: 16740211

[9] Benmachiche A Benbouhedja S Zoghmar A Humaidan P . Low LH level on the day of gnRH agonist trigger is associated with reduced ongoing pregnancy and live birth rates and increased early miscarriage rates following IVF/ICSI treatment and fresh embryo transfer. Front Endocrinol (Lausanne). (2019) 10:639. doi: 10.3389/fendo.2019.00639, PMID: 31620091 PMC6759793

[10] Balasch J Fábregues F . Is luteinizing hormone needed for optimal ovulation induction? Curr Opin Obstet Gynecol. (2002) 14:265–74. doi: 10.1097/00001703-200206000-00004, PMID: 12032381

[11] Copperman AB Benadiva C . Optimal usage of the GnRH antagonists: a review of the literature. Reprod Biol Endocrinol. (2013) 11:20. doi: 10.1186/1477-7827-11-20, PMID: 23496864 PMC3618003

[12] Hua L Wang C . Recombinant-luteinzing hormone supplementation in women during IVF/ICSI cycles with GNRH-antagonist protocol: A systematic review and meta-analysis. Eur J Obstet Gynecol Reprod Biol. (2023) 283:43–8. doi: 10.1016/j.ejogrb.2024.11.012, PMID: 36764035

[13] Geng Y Lai Q Xun Y Jin L . The effect of premature luteinizing hormone increases among high ovarian responders undergoing a gonadotropin-releasing hormone antagonist ovarian stimulation protocol. Int J Gynaecol Obstet. (2018) 142:97–103. doi: 10.1002/ijgo.12485, PMID: 29542120

[14] Kolibianakis EM Albano C Kahn J Camus M Tournaye H Van Steirteghem AC . Exposure to high levels of luteinizing hormone and estradiol in the early follicular phase of gonadotropin-releasing hormone antagonist cycles is associated with a reduced chance of pregnancy. Fertil Steril. (2003) 79:873–80. doi: 10.1016/s0015-0282(02)04920-8, PMID: 12749423

[15] Gardner DK Schoolcraft WB . Culture and transfer of human blastocysts. Curr Opin Obstet Gynecol. (1999) 11:307–11. doi: 10.1097/00001703-199906000-00013, PMID: 10369209

[16] Liu C Jiang H Zhang W Yin H . Double ovarian stimulation during the follicular and luteal phase in women ≥38 years: a retrospective case-control study. Reprod BioMed Online. (2017) 35:678–84. doi: 10.1016/j.rbmo.2017.08.019, PMID: 29030068

[17] Zhang J Du M Wu Y Wei Z Guan Y . Effect of serum progesterone levels on hCG trigger day on pregnancy outcomes in GnRH antagonist cycles. Front Endocrinol (Lausanne). (2022) 13:982830. doi: 10.3389/fendo.2022.982830, PMID: 36246920 PMC9554087

[18] Nagin DS Odgers CL . Group-based trajectory modeling in clinical research. Annu Rev Clin Psychol. (2010) 6:109–38. doi: 10.1146/annurev.clinpsy.121208.131413, PMID: 20192788

[19] Nguena Nguefack HL Pagé MG Katz J Vanasse A Dorais M Samb OM . Trajectory modelling techniques useful to epidemiological research: A comparative narrative review of approaches. Clin Epidemiol. (2020) 12:1205–22. doi: 10.2147/CLEP.S265287, PMID: 33154677 PMC7608582

[20] Casarini L Santi D Brigante G Simoni M . Two hormones for one receptor: evolution, biochemistry, actions, and pathophysiology of LH and hCG. Endocr Rev. (2018) 39:549–92. doi: 10.1210/er.2018-00065, PMID: 29905829

[21] Hillier SG Whitelaw PF Smyth CD . Follicular oestrogen synthesis: the 'two-cell, two-gonadotrophin' model revisited. Mol Cell Endocrinol. (1994) 100:51–4. doi: 10.1016/0303-7207(94)90278-x, PMID: 8056158

[22] Hsueh AJ Kawamura K Cheng Y Fauser BC . Intraovarian control of early folliculogenesis. Endocr Rev. (2015) 36:1–24. doi: 10.1210/er.2014-1020, PMID: 25202833 PMC4309737

[23] La Marca A Longo M Sighinolfi G Grisendi V Imbrogno MG Giulini S . New insights into the role of LH in early ovarian follicular growth: a possible tool to optimize follicular recruitment. Reprod BioMed Online. (2023) 47:103369. doi: 10.1016/j.rbmo.2023.103369, PMID: 37918055

[24] La Marca A Longo M . Extended LH administration as a strategy to increase the pool of recruitable antral follicles in hypothalamic amenorrhea: evidence from a case series. Hum Reprod. (2022) 37:2655–61. doi: 10.1093/humrep/deac195, PMID: 36107111

[25] Zhang D Zhang D Sun Z Deng C Yu Q Zhen J . The effect of a transient premature luteinizing hormone surge without elevated serum progesterone on *in vitro* fertilization outcomes in a gonadotropin-releasing hormone antagonist flexible protocol. Gynecol Endocrinol. (2020) 36:550–3. doi: 10.1080/09513590.2019.1683730, PMID: 31829082

[26] Gao F Wang Y Wu D Fu M Zhang Q Ren Y . A premature rise of luteinizing hormone is associated with a reduced cumulative live birth rate in patients ≥37 years old undergoing gnRH antagonist *in vitro* fertilization cycles. Front Endocrinol (Lausanne). (2021) 12:722655. doi: 10.3389/fendo.2021.722655, PMID: 34925227 PMC8678590

[27] Zhang LJ Liu D Xu LQ Wei JY Fan L Zhang XQ . Impact of luteinizing hormone on IVF/ICSI assisted reproduction on the initiation day of gonadotropin-releasing hormone antagonist protocol. Endocr Metab Immune Disord Drug Targets. (2025) 25:400–10. doi: 10.2174/0118715303281640240722070348, PMID: 39082176

[28] Liu Z Wang KH . Effect of basal luteinizing hormone (bLH) level on *in vitro* fertilization/intra-cytoplasmic injections (IVF/ICSI) outcomes in polycystic ovarian syndrome (PCOS) patients. BMC Pregnancy Childbirth. (2023) 23:618. doi: 10.1186/s12884-023-05944-4, PMID: 37644399 PMC10466855

[29] Wang B Li Z . Hypersecretion of basal luteinizing hormone and an increased risk of pregnancy loss among women with polycystic ovary syndrome undergoing controlled ovarian stimulation and intrauterine insemination. Heliyon. (2023) 9:e16233. doi: 10.1016/j.heliyon.2023.e16233, PMID: 37234655 PMC10205630

[30] Wei Y Luan T Shen J Zhang J Zhang J Su Y . LH on GnRH-ant day to basal LH affects the IVF/ICSI outcome of PCOS women undergoing GnRH-antagonist protocol. Int J Gynaecol Obstet. (2024) 164:624–32. doi: 10.1002/ijgo.15131, PMID: 37724009

[31] Wang J Ding J Qu B Zhang Y Zhou Q . Does serum LH level influence IVF outcomes in women with PCOS undergoing gnRH-antagonist stimulation: A novel indicator. J Clin Med. (2022) 11:4670. doi: 10.3390/jcm11164670, PMID: 36012922 PMC9410231

[32] Li Q Zhou X Ye B Tang M Zhu Y . Ovarian response determines the luteinizing hormone suppression threshold for patients following the gonadotrophin releasing hormone antagonist protocol: A retrospective cohort study. Heliyon. (2024) 10:e23933. doi: 10.1016/j.heliyon.2023.e23933, PMID: 38187350 PMC10767281

[33] Zhang Q Zhang K Gao Y He S Meng Y Ming L . Effect of LH level on HCG trigger day on clinical outcomes in patients with diminished ovarian reserve undergoing GnRH-antagonist protocol. Reprod Biol Endocrinol. (2024) 22:107. doi: 10.1186/s12958-024-01280-0, PMID: 39175038 PMC11340131

[34] Chen Y Li Y Li X Liu L Liu Z Gui W . Lower serum LH level was related to poor embryo quality and adverse pregnancy outcomes in fixed GnRH antagonist protocol with estradiol pretreatment. Gynecol Endocrinol. (2024) 40:2409147. doi: 10.1080/09513590.2024.2409147, PMID: 39360455

[35] Xu Y Chen J Zhang Y Qi Q Zhou J Zhou Q . Women undergoing *in vitro* fertilization/intracytoplasmic sperm injection-embryo transfer (IVF/ICSI-ET) might benefit from maintaining serum luteinizing hormone levels: A retrospective analysis. Drug Discov Ther. (2023) 17:95–103. doi: 10.5582/ddt.2022.01110, PMID: 37081647

[36] Talari K Goyal M . Retrospective studies - utility and caveats. J R Coll Phys Edinb. (2020) 50:398–402. doi: 10.4997/jrcpe.2020.409, PMID: 33469615

