# Supplementary material for: Serum luteinizing hormone trajectories during ovarian stimulation and their outcomes of IVF/ICSI: a retrospective cohort study
Source: Front Endocrinol (Lausanne). 2025 Dec 9;16:1688867. doi: 10.3389/fendo.2025.1688867 (PMC12722849; doi:10.3389/fendo.2025.1688867)
Supplement: Supplementary file 1 [file Table1.docx]

Supplementary table 1 Group based trajectories building to identify LH trajectories during ovarian stimulation

| Number of groups | order | AIC | BIC | AvePP, % | No. of participants | % Participants | Posterior probabilities >0.7 (%) |
| --- | --- | --- | --- | --- | --- | --- | --- |
| 1 | 3 | - 32389.48 | - 32404.24 | 100 | 2716 | 100 | 100 |
| 2 | 3-3 | - 31275.27 | - 31304.81 | 99.5/94.4 | 2574/141 | 94.8/5.2 | 99.5/90.1 |
| 3 | 3-3-3 | - 31041.59 | - 31085.89 | 98.1/86.9/95.2 | 2448/226/42 | 90.1/8.3/1.5 | 98.0/83.2/88.1 |
| **3a** | **3-3-2** | **- 31049.39** | **- 31090.73** | **98.3/86.5/94.6** | **2464/206/46** | **90.2/8.2/1.7** | **98.5/82.5/89.1** |
| 4 | 3-3-3-3 | - 30955.41 | - 31014.48 | 99.0/86.7/88.9/0 | 2534/112/70/0 | 93.0/4.6/2.5/0 | 99.0/75.9/82.9/0 |

LH, luteinizing hormone; AIC, akaike information criterion; BIC, bayesian information criteria; AvePP, Average Posterior Probability; No., number.

| Supplementary table 2 The cycle and embryo characteristics of patients on different LH trajectories during ovarian stimulation with posterior probabilities > 70%, n=2,639 | | | | | |
| --- | --- | --- | --- | --- | --- |
|  | Total patients | Persistently low | From middle to high | Up and down | P value |
| n | 2639 | 2428 | 170 | 41 |  |
| Total dose of Gn (IU), mean (SD) | 1980.9 | 2009.1 (986.8) ^*. #^ | 1675.1 ^*^ | 1577.5 ^#^ | **<0.001** |
| Duration of ovarian stimulation (days), mean (SD) | 9.9 (2.0) | 9.9 (2.0) | 9.8 (1.7) | 9.9 (3.5) | 0.57 |
| MII oocytes rate, % (n) | 88.0 (30987) | 88.0 (27748) | 87.5 (2610) | 88.8 (719) | 0.53 |
| 2PN fertilized rate, % (n) | 73.7 (22831) | 73.9 (20498) ^*^ | 71.6 (1868) ^*^ | 73.9 (465) | **0.04** |
| Embryo formation rate, % (n) | 74.9 (23208) | 75.0 (20800) | 73.8 (1926) | 76.6 (482) | 0.25 |
| Good-quality day-3 embryo rate, % (n) | 40.2 (12445) | 40.2 (11155) ^*^ | 40.5 (1058) ^*, #^ | 36.9 (232) ^*, #^ | **0.03** |
| Blastocyst culture, n | 2303 | 2113 | 157 | 33 |  |
| Blastocyst formation rate, % (n) | 51.5 (8886) | 51.5 (7937) | 52.0 (778) | 48.6 (171) | 0.51 |
| Good-quality blastocyst formation rate, % (n) | 37.3 (6435) | 37.5 (5773) | 36.7 (550) | 31.8 (112) | 0.09 |
| Elevated serum levels of P on trigger day, % (n) | 10.6 (229) | 10.2 (200) ^*, #^ | 13.2 (20) ^*^ | 25.7 (9) ^#^ | **<0.001** |

Abbreviations: Gn, gonadotropin-releasing hormone; n, number; SD, standard deviation; MII, metaphase II; PN, 2 pronuclear; ET, embryo transfer; P, progesterone.

^*, #^ Same superscript in a row indicates statistically significant differences.

| Supplementary table 3 Association of LH trajectories with IVF/ICSI and embryo outcomes with posterior probabilities > 70% | | | |
| --- | --- | --- | --- |
|  |  | Unadjusted | Adjusted ^*^ |
| LH trajectory group | % (n) | β (95% CI) | β (95% CI) |
| **No. of oocytes received** | | | |
| Persistently low | 13.0 (7.8) | Reference | Reference |
| From middle to high | 17.6 (10.4) | **4.57 (2.98 to 6.15)** | **4.27 (2.72 to 5.83)** |
| Up and down | 17.3 (10.9) | **4.28 (0.96 to 7.61)** | **3.94 (0.51 to 7.37)** |
| **No. of MII oocytes** | | | |
| Persistently low | 11.4 (7.0) | Reference | Reference |
| From middle to high | 15.4 (9.1) | **3.92 (2.53 to 5.32)** | **3.66 (2.29 to 5.03)** |
| Up and down | 15.3 (10.1) | **3.91 (0.83 to 6.70)** | **3.61 (0.43 to 6.79)** |
| **No. of 2PN fertilized oocytes** | | | |
| Persistently low | 8.4 (5.6) | Reference | Reference |
| From middle to high | 11.0 (7.1) | **2.55 (1.46 to 3.63)** | **2.37 (1.30 to 3.44)** |
| Up and down | 11.3 (7.8) | **2.90 (0.53 to 5.27)** | **2.70 (0.27 to 5.12)** |
| **No. of day-3 embryos** | | | |
| Persistently low | 8.6 (5.7) | Reference | Reference |
| From middle to high | 11.3 (7.3) | **2.76 (1.64 to 3.88)** | **2.58 (1.47 to 3.69)** |
| Up and down | 11.8 (8.1) | **3.19 (0.72 to 5.66)** | **2.98 (0.46 to 5.51)** |
| **No. of good-quality day-3 embryos** | | | |
| Persistently low | 4.6 (4.1) | Reference | Reference |
| From middle to high | 6.2 (5.2) | **1.63 (0.83 to 2.43)** | **1.53 (0.73 to 2.33)** |
| Up and down | 5.7 (5.8) | 1.06 (-0.69 to 2.82) | 0.95 (-0.80 to 2.71) |
| **No. of blastocyst culture** | | | |
| Persistently low | 7.3 (5.3) | Reference | Reference |
| From middle to high | 9.5 (6.6) | **2.24 (1.19 to 3.30)** | **2.10 (1.06 to 3.15)** |
| Up and down | 10.7 (7.3) | **3.38 (0.91 to 5.84)** | **3.25 (0.75 to 5.74)** |
| **No. of blastocyst** | | | |
| Persistently low | 3.8 (3.6) | Reference | Reference |
| From middle to high | 5.0 (4.2) | **1.20 (0.53 to 1.87)** | **1.14 (0.48 to 1.81)** |
| Up and down | 5.2 (4.3) | 1.43 (-0.04 to 2.89) | 1.37 (-0.10 to 2.85) |
| **No. of good-quality blastocyst** | | | |
| Persistently low | 2.7 (3.0) | Reference | Reference |
| From middle to high | 3.5 (3.3) | **0.77 (0.24 to 1.30)** | **0.74 (0.21 to 1.27)** |
| Up and down | 3.4 (2.9) | 0.66 (-0.33 to 1.65) | 0.63 (-0.37 to 1.63) |

Abbreviations: n or No., number; CI, confidence interval; MII, metaphase II; 2PN, 2 pronuclear; AFC, antral follicle counting; BMI, body mass index.

^*^ Adjusted for age and BMI.

| Supplementary table 4 Pregnancy outcomes among patients underwent fresh embryo transfer cycle with different LH trajectories during ovarian stimulation with posterior probabilities > 70% | | | | | |
| --- | --- | --- | --- | --- | --- |
|  | Total patients | Persistently low | From middle to high | Up and down | *P* value |
| All Embryo transfer, n | 686 | 656 | 24 | 6 |  |
| Biochemical pregnancy rate, % (n) | 58.3 (400) | 58.7 (385) | 54.2 (13) | 33.3 (2) | 0.42 |
| Clinical pregnancy rate, % (n) | 47.5 (326) | 47.9 (314) | 41.7 (10) | 33.3 (2) | 0.66 |
| Uterine pregnancy rate, % (n) | 97.6 (318) | 97.5 (306) | 100.0 (10) | 100.0 (2) | 0.99 |
| Blastocyst transfer, n | 567 | 541 | 21 | 5 |  |
| Biochemical pregnancy rate, % (n) | 60.0 (340) | 60.6 (328) | 52.4(11) | 20.0 (1) | 0.14 |
| Clinical pregnancy rate, % (n) | 49.2 (279) | 49.9 (270) | 38.1 (8) | 20.0 (1) | 0.24 |
| Uterine pregnancy rate, % (n) | 97.5 (272) | 97.4 (263) | 100.0 (10) | 100.0 (2) | 0.99 |
| Cleavage embryo transfer, n | 119 | 115 | 3 | 1 |  |
| Biochemical pregnancy rate, % (n) | 50.4 (60) | 49.6 (57) | 66.7 (2) | 100.0 (1) | 0.51 |
| Clinical pregnancy rate, % (n) | 39.5 (47) | 38.3 (44) | 66.7 (2) | 100.0 (1) | 0.28 |
| Uterine pregnancy rate, % (n) | 97.9 (46) | 97.7 (43) | 100.0 (2) | 100.0 (1) | 0.97 |

Abbreviations: n, number; LH, luteinizing hormone.

| Table 5 Association of LH trajectories with pregnancy outcomes among patients underwent fresh blastocyst transfer with posterior probabilities > 70% | | |
| --- | --- | --- |
|  | Unadjusted | Adjusted ^*^ |
| LH trajectory group | RR (95% CI) | RR (95% CI) |
| **Biochemical pregnancy** |  |  |
| Persistently low | Reference | Reference |
| From middle to high | 0.87 (0.57-1.31) | 0.86 (0.57-1.29) |
| Up and down | 0.33 (0.51-1.91) | 0.31 (0.05-1.80) |
| **Clinical pregnancy** |  |  |
| Persistently low | Reference | Reference |
| From middle to high | 0.76 (0.44-1.33) | 0.76 (0.44-1.33) |
| Up and down | 0.40 (0.07-2.32) | 0.38 (0.07-2.21) |

Abbreviations: RR, relative risk; CI, confidence interval; AFC, antral follicle counting; BMI, body mass index.

^*^ Adjusted for age and BMI.
